# ErbB2-Dependent Chemotaxis Requires Microtubule Capture and Stabilization Coordinated by Distinct Signaling Pathways

**DOI:** 10.1371/journal.pone.0055211

**Published:** 2013-01-29

**Authors:** Khedidja Benseddik, Nadine Sen Nkwe, Pascale Daou, Pascal Verdier-Pinard, Ali Badache

**Affiliations:** 1 Centre de Recherche en Cancérologie de Marseille, Inserm U1068, Marseille, France; 2 Institut Paoli-Calmettes, Marseille, France; 3 Aix-Marseille Univ, Marseille, France; 4 CNRS, UMR7258, Marseille, France; King's College London, United Kingdom

## Abstract

Activation of the ErbB2 receptor tyrosine kinase stimulates breast cancer cell migration. Cell migration is a complex process that requires the synchronized reorganization of numerous subcellular structures including cell-to-matrix adhesions, the actin cytoskeleton and microtubules. How the multiple signaling pathways triggered by ErbB2 coordinate, in time and space, the various processes involved in cell motility, is poorly defined. We investigated the mechanism whereby ErbB2 controls microtubules and chemotaxis. We report that activation of ErbB2 increased both cell velocity and directed migration. Impairment of the Cdc42 and RhoA GTPases, but not of Rac1, prevented the chemotactic response. RhoA is a key component of the Memo/ACF7 pathway whereby ErbB2 controls microtubule capture at the leading edge. Upon Memo or ACF7 depletion, microtubules failed to reach the leading edge and cells lost their ability to follow the chemotactic gradient. Constitutive ACF7 targeting to the membrane in Memo-depleted cells reestablished directed migration. ErbB2-mediated activation of phospholipase C gamma (PLCγ) also contributed to cell guidance. We further showed that PLCγ signaling, via classical protein kinases C, and Memo signaling converged towards a single pathway controlling the microtubule capture complex. Finally, inhibiting the PI3K/Akt pathway did not affect microtubule capture, but disturbed microtubule stability, which also resulted in defective chemotaxis. PI3K/Akt-dependent stabilization of microtubules involved repression of GSK3 activity on the one hand and inhibition of the microtubule destabilizing protein, Stathmin, on the other hand. Thus, ErbB2 triggers distinct and complementary pathways that tightly coordinate microtubule capture and microtubule stability to control chemotaxis.

## Introduction

Aberrant activation of receptor tyrosine kinases contribute to tumor development in various cancers including colorectal, lung, head and neck, brain and breast. Overexpression of the ErbB2/Her2/Neu receptor is observed in 20 to 25% of breast cancer patients and is associated with a poor prognosis [1]. While the role of ErbB2 in tumor cell proliferation and survival has been largely studied, less is known about the possible contribution of ErbB2 to tumor cell motility, invasion and metastasis [2].

ErbB2 is part of the ErbB family of receptor tyrosine kinase which includes ErbB1/Her1/EGFR (epidermal growth factor receptor), ErbB3 and ErbB4 [3]. ErbB2 has no known ligand. However, ligand binding to other ErbB family members triggers formation of receptor homodimers and ErbB2-containing heterodimers. In fact, ErbB2 containing dimers were found to be the most effective in term of signaling and transformation ability [4], [5]. Receptor dimerization leads to kinase activation and phosphorylation of specific tyrosine residues within the receptor C-terminal tail. Phosphorylated tyrosines serve as docking sites for PTB or SH2 domain containing proteins which initiate a variety of signaling pathways including the Ras/MAPK, Akt/PI3K, p38MAPK, JNK, STAT and Src-dependent pathways [6]. While many signaling pathways have been involved in cell motility, we observed little redundancy among major ErbB2-induced signaling pathways, indicating that each pathway carries a unique function [7].

Cell motility is a complex process which integrates numerous discrete subcellular events that must be coordinated in space and time [8]. Schematically, in response to a ligand, cells polarize in the direction of the stimulus and undergo morphogenetic changes. The formation of a dense actin network underneath the plasma membrane causes membrane protrusion. Assembly and maturation of adhesion complexes organized around integrins allow cell attachment to the extracellular matrix. Contraction of the acto-myosin stress fibers, which are anchored at adhesion sites, favors the detachment of the rear end and progression of the cell body. These processes are under the control of RhoGTPases. Cell motility is also accompanied by the assembly, at microtubule organizing centers (MTOCs), of microtubules whose dynamic plus-ends explore protrusions until reaching stabilizing structures at the cell periphery. Nonetheless, the function of microtubules during cell motility is still debated [9].

We have previously identified a signaling pathway whereby ErbB2 controls microtubule capture at the cell leading edge. This pathway involves the recruitment of RhoA to the plasma membrane via the Memo adaptor and the activation of its effector, mDia1/DRF1 [7], [10]. In turn, activation of mDia1 results in repression of GSK3 activity, allowing localization of APC and ACF7 to the cell membrane and ACF7-dependent microtubule capture [11]. However, it is not clear how the numerous intracellular pathways triggered by ErbB2 receptor activation are coordinated to control microtubule outgrowth and stabilization in cell protrusions and how they might influence directed cell migration.

In this study we characterized the signaling network downstream of ErbB2 that underlies microtubule capture and stabilization in cell protrusions and its contribution to the cell chemotactic response.

## Results

### ErbB2-dependent chemotaxis

**Figure 1:**
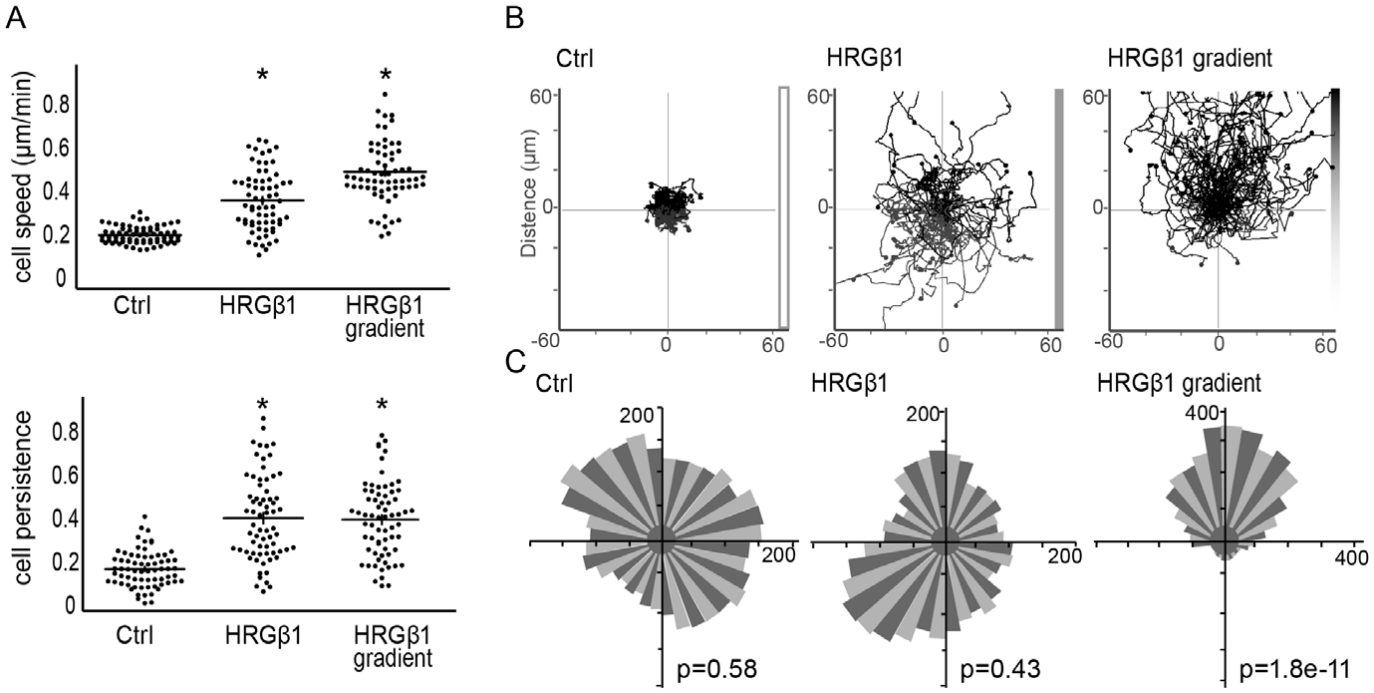
HRG-induced ErbB2 activation triggers a chemotactic response. (**A**) T47D cells plated in Dunn chambers were tracked by time lapse microscopy over 4 h in the absence (Ctrl) or in the presence of a homogenous concentration of HRG or a HRG gradient (upwards). (**B**) Individual tracks of migrating cells were plotted. Cell velocity and persistence calculated. 90–150 cells were tracked in three independent experiments; mean+/−s.e.m. is shown; * p<0.01. (**C**) To visualize cell orientation independently of cell speed, data were plotted as Rose diagrams which indicated the distribution of migration angles. The Rayleigh test evaluates unimodal distribution of cell directions at end points. p>0.05 is considered uniform distribution (random migration).

In order to determine the contribution of ErbB2-induced signaling to the discrete events required for cell motility, we have set up an ErbB2-dependent chemotactic migration assay in Dunn chambers. Motility of the T47D and SKBr3 breast carcinoma cell lines was evaluated by tracking individual cells as they migrated between two chambers in response to heregulin β1 (HRG; Fig. 1A). It was shown previously that HRG-induced motility was strictly dependent on ErbB2 [7], [12]. Addition of equal concentrations of HRG in both chambers increased T47D (Fig. 1B) and SKBr3 (data not shown) cell speed and persistence. When exposed to a gradient of HRG, cell speed was further increased (Fig. 1B) and a large majority of cell migrated towards the source of HRG (Fig. 1A); this was also clearly visible in Rose plots (Fig. 1C) that reflected cell distribution independently of the distance traveled. The Rayleigh statistical test confirmed that, in the absence of ligand or in the presence of homogenous concentrations of HRG, cells showed random migration (p>0.05), while in the presence of the HRG gradient, migration was very strongly biased towards the areas of highest HRG concentrations.

### Role of the Memo/RhoA/ACF7 pathway in ErbB2-dependent chemotaxis

**Figure 2:**
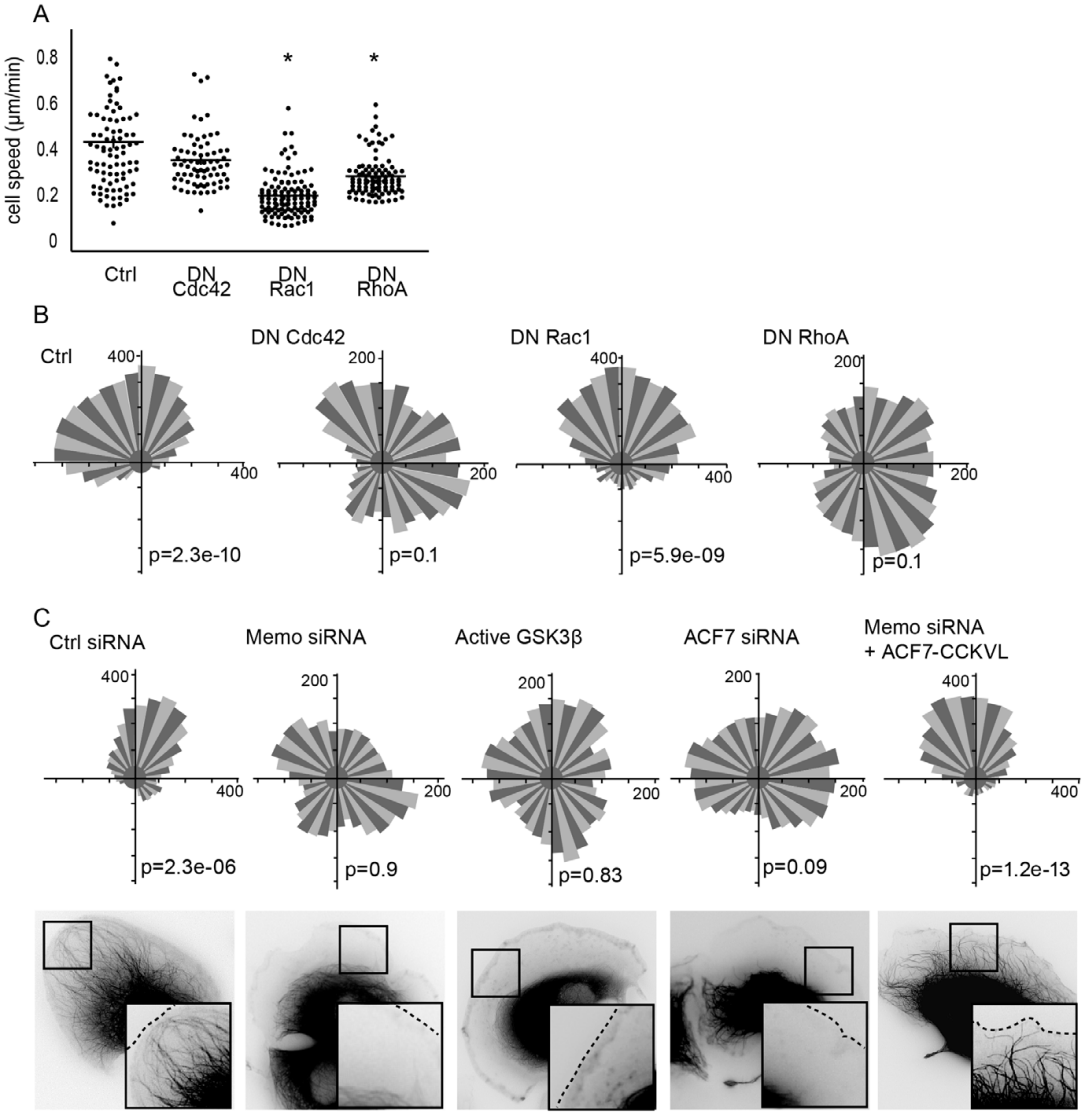
Role of Rho signaling in ErbB2-dependent chemotaxis. Chemotaxis of T47D cells expressing dominant negative (DN) RhoA, Rac1 or Cdc42 or a control vector (Ctrl) in response to a HRG gradient was analyzed as described in Fig. 1. (**A**) Cell velocity was calculated from individual tracks; mean+/−s.e.m. is shown; * p<0.01. (**B**) Rose diagrams and Rayleigh tests; p>0.05 is considered uniform distribution. Cdc42 and RhoA are involved in oriented migration. (**C**) Implication of the Memo/GSK3/ACF7 signaling pathway in chemotaxis. Directional distribution (upper panels) of cells expressing control (Ctrl), Memo or ACF7 siRNA, or active GSK3 or membrane targeted ACF7 (ACF7-CCKVL) constructs was evaluated. (Lower panels) Still images of EGFP-tubulin expressing cells, analyzed by time-lapse fluorescence microscopy 30 min after addition of HRG, are shown. Insets: zooms showing the presence or absence of peripheral microtubules in cell protrusions.

RhoGTPases are amongst the main regulators of cell migration. In order to evaluate the contribution of RhoGTPases to ErbB2-dependent chemotaxis, cells were transfected with dominant negative (DN) Rho constructs, before being exposed to the HRG gradient. Inhibition of Rac1 activity led to disturbed cell protrusion (data not shown) and to a significant decrease in cell speed (Fig. 2A), as expected from the role Rac1 plays in cortical actin formation. Interestingly, even though cell velocity was considerably reduced, cells were still capable of gradient sensing and chemotactic migration (Fig. 2B). Cdc42 was previously involved in both actin nucleation and cell polarity. Inhibition of Cdc42 activity did not significantly affect cell speed (Fig. 2A), but prevented oriented migration (Fig. 2B). DN-RhoA significantly affected cell speed in accordance with RhoA function in cell contractility (Fig. 2A). Unexpectedly, inhibition of RhoA activity also led to a complete loss of chemotaxis (Fig. 2B).

We have previously identified a signaling pathway whereby ErbB2 controls microtubule capture at the cell leading edge, which involved recruitment of RhoA to the plasma membrane via the Memo adaptor, repression of GSK3 activity and re-localization of ACF7 to the cell membrane, via APC [10], [11]. Similarly, in the chemotaxis setting RhoA was recruited to cortical areas and co-localized with its effectors, mDia1 and APC (data not shown). Therefore, we have investigated whether the loss of oriented migration observed upon inhibition of RhoA activity was linked to disturbance of the Memo/ACF7 pathway. SiRNA-mediated depletion of Memo or ACF7 (supplementary material Fig. S1A,B) or expression of a constitutively active form of GSK3 strongly disturbed the chemotactic response, leading to random cell motility (Fig. 2C). Loss of chemotaxis paralleled defective capture of microtubule at the cell leading edge (Fig. 2C). We have previously shown that expression of an ACF7 mini-gene composed of ACF7 N-terminal and C-terminal sequences, rescued microtubule capture in Memo knockdown cells only when fused to the CCKVL membrane targeting sequence [11]. We confirmed that expression of ACF7-CCKVL restored peripheral microtubules; but we also showed that it was sufficient to reestablish oriented migration in both T47D (Fig. 2C) and SKBr3 (data not shown) Memo-depleted cells. Thus, our data show that the Memo/ACF7 pathway is required for ErbB2-induced chemotactic migration and link microtubule capture at the cell front to oriented migration.

### Characterization of the PLCγ pathway involved in ErbB2-dependent chemotaxis

Activated ErbB2 triggers multiple pathways that are involved in cell motility. Previous data has shown that PLCγ was involved in microtubule outgrowth and cell motility [7], [13]. However, the mechanism whereby PLCγ controlled microtubule capture was not explored.

**Figure 3:**
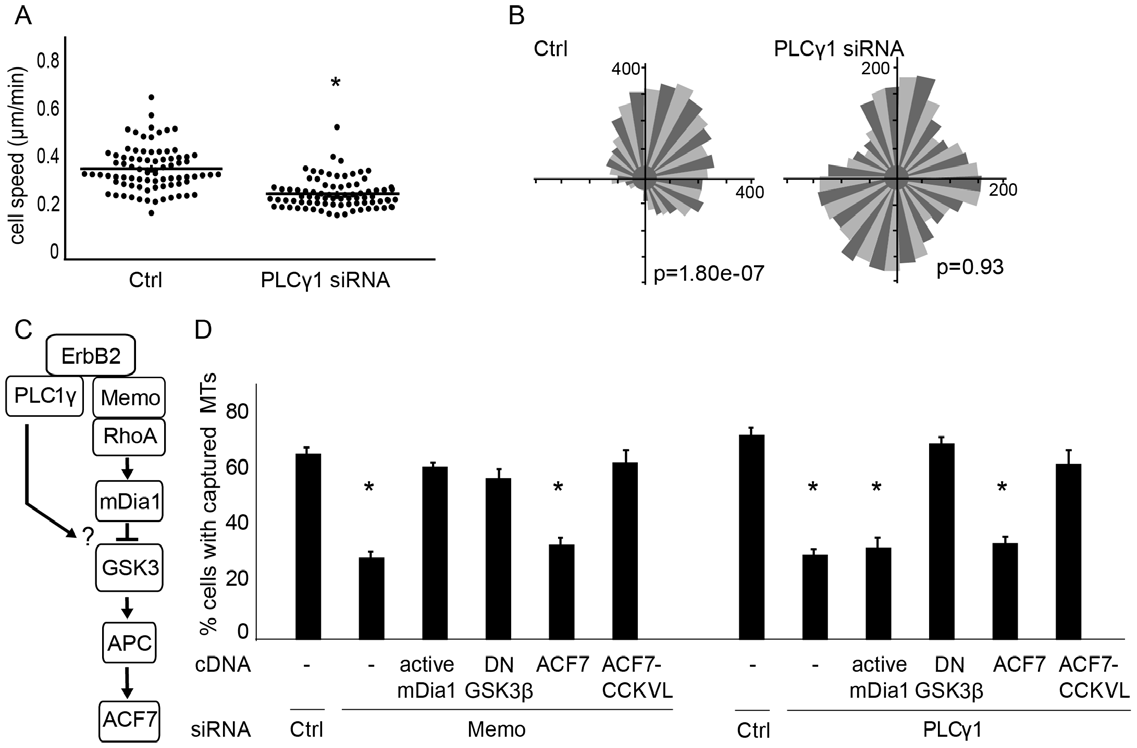
PLCγ contributes to microtubule capture and chemotaxis; convergence with the Memo signaling pathway. Velocity of SKBr3 cells transfected with control or PLCγ siRNA in response to a HRG gradient was assayed as described in Fig. 1. (**A**) Cell velocity was calculated from individual tracks; mean+/−s.e.m. is shown; * p<0.01. (**B**) Rose diagrams and Rayleigh tests; p>0.05 is considered random migration. (**C**) Schematic of the Memo/ACF7 pathway. (**D**) EGFP-tubulin expressing cells were visualized by time-lapse microscopy (see illustrations in supplementary material Fig. S2) and the percentage of cells with peripheral microtubules (MTs) was evaluated 30 min after addition of HRG. Cells were transfected with Memo, PLCγ or control (Ctrl) siRNA, together with active mDia1, dominant negative GSK3 (DN-GSK3), ACF7 minigene (ACF7) or membrane targeted ACF7 minigene (ACF7-CCKVL). 90–150 cells were counted per condition in three independent experiments; mean+/−s.e.m. is shown; * p<0.01. Expression of DN-GSK3 or membrane targeted ACF7 rescued microtubules in both Memo and PLCγ-depleted cells, while active mDia1 restored microtubules only in Memo-depleted cells.

Using siRNAs (supplementary material Fig. S1C), we confirmed that depletion of PLCγ had a significant impact on cell velocity (Fig. 3A), chemotactic migration (Fig. 3B) and microtubule capture (Fig. 3D; supplementary material Fig. S2). We next investigated if the PLCγ and Memo/ACF7 signaling pathways interacted. Similarly to what was observed in Memo-depleted cells, expression of membrane-targeted ACF7 restored microtubule capture in both SKBr3 (Fig. 3D; supplementary material Fig. S2) and T47D (data not shown) PLCγ-depleted cells, while expression of ACF7 was not efficient. These results suggested that, similarly to Memo, PLCγ controlled microtubule capture via the recruitment of ACF7 to the leading edge. To determine at what level the two pathways converged, we expressed components of the Memo signaling cascade in either Memo- or PLCγ-depleted cells (Fig. 3C): overexpression of constitutively active mDia1 or of DN-GSK3 restored functionality of the Memo pathway in Memo-knockdown cells (Fig. 3D and supplementary material Fig. S2). In contrast, while DN-GSK3 restored microtubule capture, expression of active mDia1 did not restore microtubules in PLCγ-knockdown cells (Fig. 3D and supplementary material Fig. S2), indicating that PLCγ impacted the Memo pathway downstream of mDia1. Moreover, we found that PLCγ was required for ErbB2-induced relocalization to the leading edge of phosphorylated GSK3 and APC, but not of mDia1 (supplementary material Fig. S3). Altogether, these results strongly suggest that the PLCγ pathway converges towards the Memo/ACF7 pathway, downstream of mDia1 and upstream of GSK3.

### Role of Protein Kinases C (PKCs) in ErbB2-induced microtubule capture

PLCγ is known to catalyze the hydrolysis of membrane phosphatidylinositol 4,5-bisphosphate into diacylglycerol (DAG) and inositol 1,4,5-trisphosphate, which in turn leads to the release of intracellular Ca^2+^. As a consequence PLCγ activation leads to the activation of various PKCs. The PKC family can be divided in distinct subgroups according to their regulatory domains: the conventional PKCs (cPKCs) are activated by a combination of DAG and Ca^2+^-dependent phospholipid binding; novel PKCs (nPKCs) are activated by DAG and phospholipids, but do not directly respond to Ca^2+^; the atypical PKCs (aPKCs) do not generally respond to Ca^2+^ or DAG [14].

**Figure 4:**
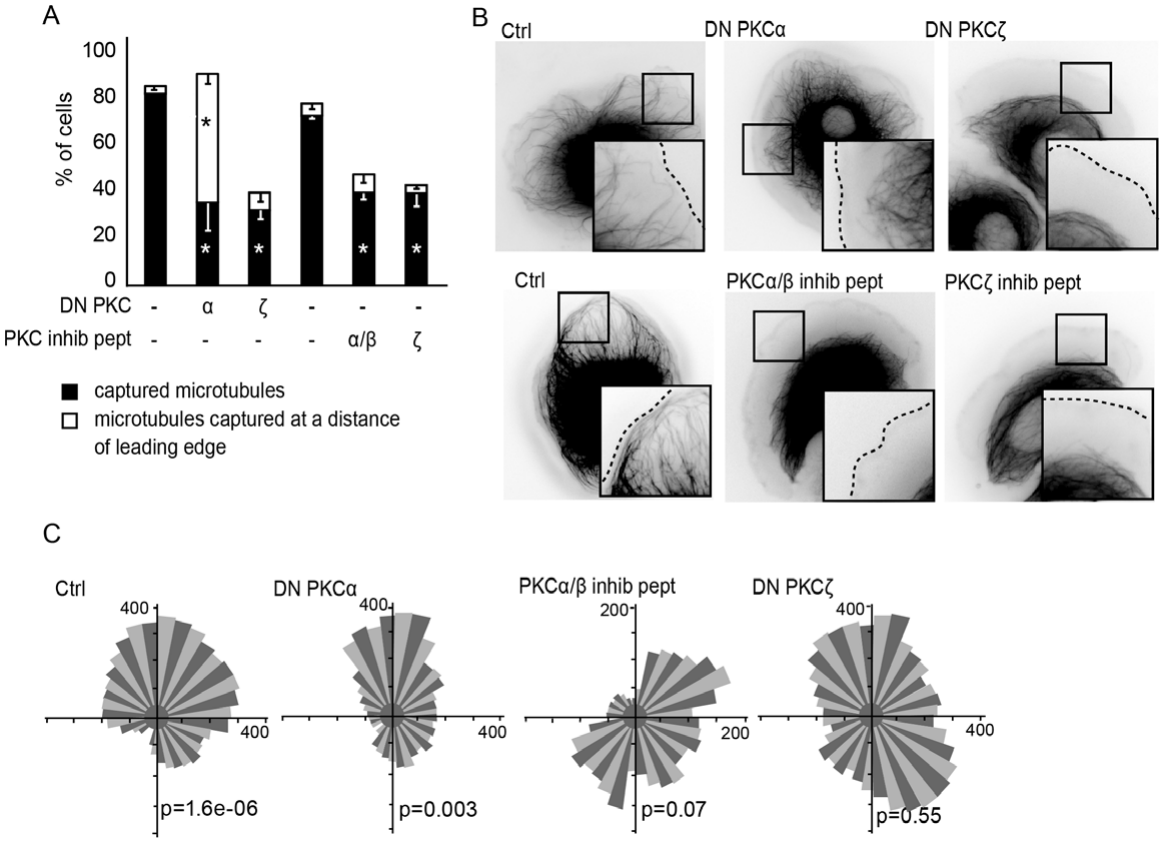
Role of PKCs in microtubule capture and chemotaxis. SKBr3 cells expressing DN-PKC constructs together with EGFP-tubulin were treated with HRG for 30 min before live fluorescence microscopy. When indicated, cells were treated with PKCα/β and PKCζ inhibitory peptides, before analysis of microtubules. (**A**) Percentage of cells with microtubules captured at the cell cortex or cells with microtubules stabilized at a short distance of the cell cortex was determined. 90–150 cells were counted per condition in three independent experiments; mean+/−s.e.m. is shown; * p<0.01 relative to the respective controls. (**B**) Still images illustrating microtubule status. (**C**) SKBr3 cells expressing EGFP-tubulin and DN-PKC constructs or PKC inhibitory peptides were assayed for chemotaxis in response to HRG. Rose diagrams and Rayleigh tests are shown; p>0.05 is considered random migration.

We have evaluated which PKCs were involved in ErbB2-dependent microtubule capture, by expressing DN PKC constructs representative of the various PKC subgroups into SKBr3 cells (supplementary material Fig. S4A). Disturbing the activity of PKCα, PKCβ, PKCγ (cPKCs), PKCδ (an nPKC) or PKCζ (an aPKC) led to abnormal peripheral microtubules, while DN constructs for the PKCε and η (nPKCs) had no effect (supplementary material Fig. S4B). Interestingly, when individual cPKCs or PKCδ were inhibited, microtubules were disturbed but showed a phenotype distinct from the one seen upon inhibition of Memo or PLCγ (Fig. 4A,B). Memo depletion typically induced microtubules that failed to extend within cell protrusions and remained at the basis of the lamella, with microtubule ends running parallel to the cell membrane; upon inhibition of individual cPKCs, microtubules were capable of extending within the lamella, pointed perpendicularly to the cell front, but remained at a short distance from the leading edge. Inhibition of PKCζ led to the typical arrest of microtubules at the basis of the lamella (Fig. 4A,B).

To confirm the data obtained with the DN PKC constructs, we have used inhibitory peptides (pseudosubstrates) specific of cPKCs, PKCη or PKCζ. Similarly, to what we observed with DN constructs, peptide-mediated inhibition of cPKCs or PKCζ activity prevented microtubule capture (Fig. 4A,B), while PKCη inhibitory peptide had no effect (data not shown). However, cPKC inhibitory peptide had a more drastic effect that inhibition of individual cPKCs via DN constructs, yielding microtubules arrested at the basis of the lamella.

We have postulated that defects in microtubule capture correlated with loss of the chemotactic response. Thus, we have investigated the consequence of PKC inhibition on oriented migration. Inhibition of cPKCs or PKCζ activity that led to lamella-arrested microtubules, also resulted in disturbed cell chemotaxis (Fig. 4C). Interestingly, cells in which PKCα activity was inhibited via the DN construct, that showed microtubules stabilized at a short distance from the leading edge, were still capable of oriented migration (Fig. 4C).

**Figure 5:**
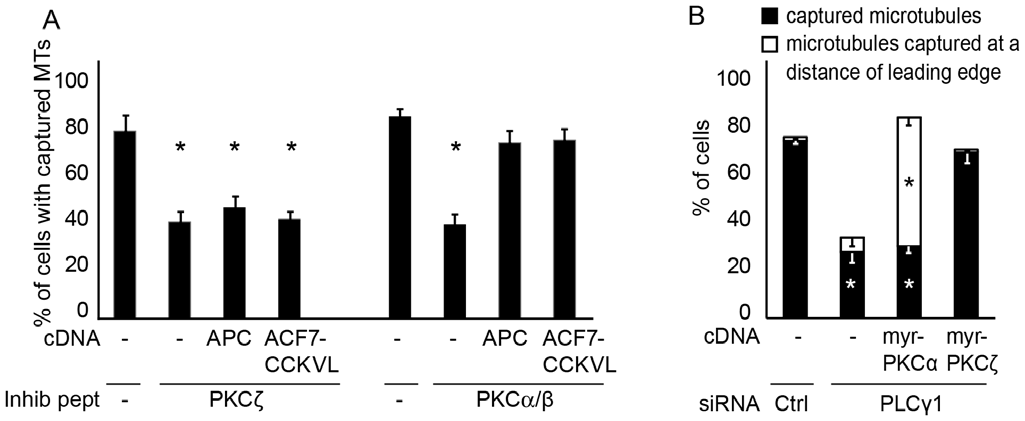
PKCs contribute to PLCγ-dependent microtubule capture. (**A**) SKBr3 cells expressing EGFP-tubulin and APC or membrane-targeted ACF7 (ACF-CCKVL) were treated with PKC inhibitory peptides, before addition of HRG and determination of the percentage of cells displaying peripheral microtubules by live fluorescence microscopy. Expression of APC or ACF7-CCKVL only rescued microtubules of cPKC-inhibited and not of aPKC-inhibited cells. (**B**) SKBr3 cells expressing EGFP-tubulin were transfected with control (Ctrl) or PLCγ siRNA and constitutively active PKCα (myr-PKCα) or PKCζ (myrPKC)ζ, before addition of HRG and analysis by live fluorescence microscopy. Percentages of cells with microtubules captured at the leading edge and cells with microtubules stabilized at a short distance of the leading edge were determined. 90–150 cells were counted per condition in three independent experiments; mean+/−s.e.m. is shown; * p<0.01 relative to the respective controls.

Finally, we explored the link between PKCs and ACF7-mediated microtubule capture. Expression of the membrane-targeted ACF7 (ACF7-CCKVL) or of APC (which allows the recruitment of ACF7 to the membrane [11]) restored the capture of microtubules in cells in which cPKC activity was inhibited (Fig. 5A; supplementary material Fig. S5A). Moreover, ErbB2-induced association of phosphorylated GSK3 and APC to the leading edge was disturbed by the cPKC inhibitory peptide, but not by individual inhibition of cPKCs via DN constructs (supplementary material Fig. S6). In contrast, expression of ACF7-CCKVL or APC failed to restore microtubules in cells in which PKCζ activity was disturbed (Fig. 5A; supplementary material Fig. S5A); interestingly, recruitment of phosphorylated GSK3, APC, mDia1, RhoA and Memo was dependent on PKCζ activity (supplementary material Fig. S6). The same results were observed in T47D cells (data not shown). These results indicate that cPKCs control microtubule capture via the recruitment of ACF7 to the plasma membrane, whereas PKCζ acts via a more complex mechanism.

Thus, both PLCγ and cPKCs mediates ErbB2-induced microtubule capture via an ACF7-dependent pathway. In order to demonstrate that cPKCs are actual effectors of PLCγ, we evaluated if a constitutively active, myristoylated, form of PKCα is able to compensate the loss of PLCγ. We observed that active PKCα restored extension of microtubules within cell protrusions (Fig. 5B and supplementary material Fig. S6B). However, similarly to what was observed upon inhibition of individual cPKCs, microtubules stabilized at a short distance of the cell membrane. In contrast, active PKCζ restored microtubules that stabilized at the leading edge (Fig. 5B and supplementary material Fig. S5B). Thus, both PKCα and PKCζ act downstream of PLCγ for ErbB2-dependent capture of microtubules, but operate via distinct mechanisms.

### The PI3K pathway controls microtubule stability and chemotaxis

**Figure 6:**
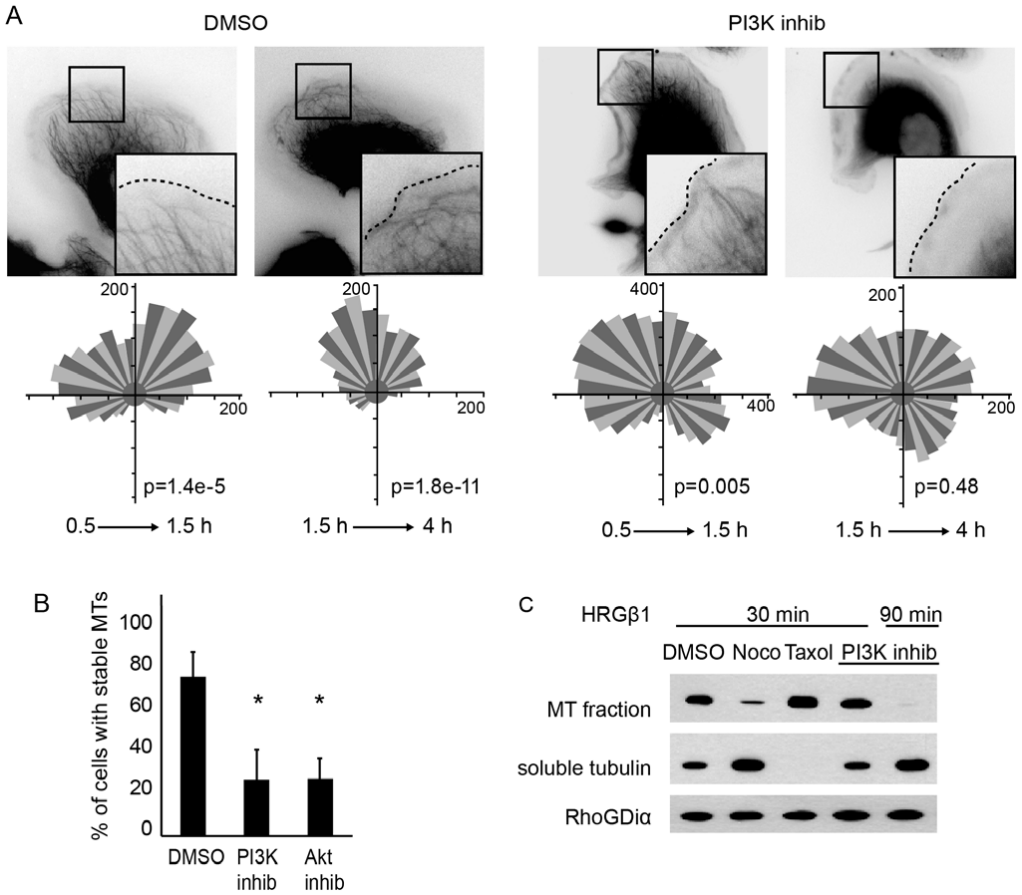
The PI3K pathway controls ErbB2- dependent microtubule stability and chemotaxis. (**A**) Upper panels: SKBr3 cells expressing EGFP-tubulin were pre-treated with DMSO or the PI3K inhibitor LY294002, before addition of HRG and analysis by time-lapse fluorescence microcopy. Still images, from Video 1, corresponding to early (0.5 to 1.5 h) and late (>1.5 h) time points are shown. Lower panels: Cells loaded on a Dunn chamber were treated as in A, for analysis of the chemotactic response at early time points (0.5 to 1.5 h) or late time points (1.5 to 4 h). Rose diagrams and Rayleigh tests are shown. p>0.05 is considered random migration. (**B**) The percentage of cells with disassembled microtubules was evaluated. 90–150 cells were counted per condition in three independent experiments; mean+/−s.e.m. is shown; * p<0.01. (**C**) Cells were pretreated with the indicated drugs, before addition of HRG for 30 or 90 min. Cells were fractionated into microtubule-enriched and soluble tubulin-enriched fractions and tubulin content was analyzed by Western blotting. An anti-RhoGDI antibody was used to assess protein loading. Nocodazole (Noco) and Taxol were used as reference microtubule-destabilizing and -stabilizing drugs, respectively.

Our results showed the involvement of the Memo and PLCγ pathways in ErbB2-dependent microtubule capture and chemotactic migration. Next, we investigated the contribution of canonical pathways to ErbB2-dependent chemotaxis. Using small molecule inhibitors, we have inhibited the Ras/MAPK, the p38MAPK and the PI3K/Akt pathways in SKBr3 cells. Surprisingly, disturbance of any of these pathways prevented oriented migration (supplementary material Fig. S7A). However, in contrast to what we observed previously, loss of chemotaxis was not associated with defective microtubule capture (supplementary material Fig. S7B). In fact, closer examination of the effect of the PI3K inhibitor, LY294004, showed that microtubules were initially captured at the leading edge, but progressively disassembled, starting from the cell periphery (Fig. 6A; supplementary material Movie 1). Ninety minutes after HRG treatment, most cells were virtually devoid of polymerized microtubules (Fig. 6B). Similar microtubule disassembly was induced by Akt inhibitor VIII (Fig. 6B), confirming that the effect was due to inhibition of the PI3K/Akt pathway. Biochemical analysis confirmed the loss of microtubules and the increase in soluble tubulin upon inhibition of PI3K (Fig. 6C) or Akt (data not shown). In contrast inhibition of the Ras/MAPK or the p38MAPK pathway had little or no impact on microtubule assembly (supplementary material Fig. S7C). Similar results were observed in T47D cells.

Our data suggest that microtubules play a critical role in oriented migration. If this assumption is correct, the inhibition of PI3K, which induced slow and progressive microtubule disassembly, should have a gradual impact on chemotaxis as well. We actually observed that at early time points, when microtubules were intact, cell migrated in an oriented manner, whereas at later time points, when microtubules were disassembled, cells lost the ability to follow the gradient, as confirmed by the Rayleigh test (Fig. 6A and video 1). This gradual disturbance of chemotactic behavior was specifically associated with inhibition of the PI3K pathway, as inhibition of Memo or PLCγ led to an immediate and persistent loss of oriented migration (data not shown).

### Mechanisms of PI3K-mediated microtubule stability

**Figure 7:**
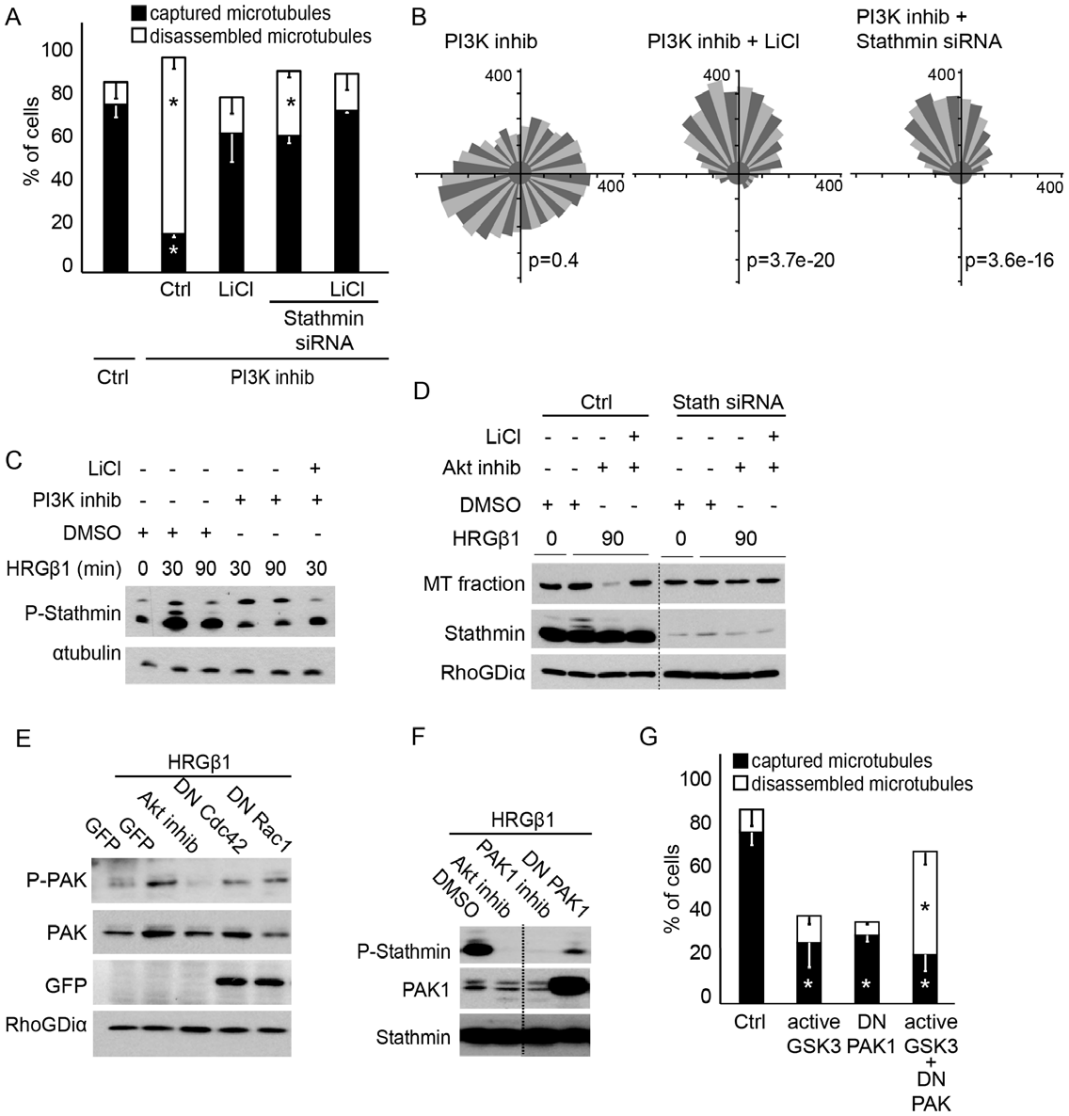
Pak1 and Stathmin act downstream of PI3K to control microtubule stability and chemotaxis. (**A**) SKBr3 cells were transfected with EGFP-tubulin and Stathmin (Stath) siRNA, and treated with LY294004 (PI3K inhib) or LiCl to inhibit GSK3 activity, as indicated, before addition of HRG. The percentage of cells with captured or disassembled microtubules was evaluated; * p<0.01 relative to the respective controls. (**B**) Cells loaded on a Dunn chamber were treated as in A, for analysis of the chemotactic response. Rose diagrams and Rayleigh tests are shown. p>0.05 is considered random migration. (**C**) T47D cells were pre-treated with the indicated inhibitor before addition of HRG. Phosphorylation of Stathmin was evaluated by Western blotting using an antibody against phosphorylated Ser16. An anti-tubulin antibody was used to assess protein loading. Phosphorylation of Stathmin is dependent on PI3K activity. (**D**) Cells were transfected with control or Stathmin siRNA, before treatment with the indicated inhibitor. After treatment with HRG, cells were fractionated and the microtubule-enriched fraction was analyzed by Western blotting using an anti-tubulin antibody. Cell lysates were also probed for Stathmin and RhoGDI (loading control). Depletion of Stathmin prevents microtubule disassembly induced by the Akt inhibitor. (**E**) Cells were transfected with DN-Rac1, DN-Cdc42 or pre-treated with the Akt inhibitor, before addition of HRG and analysis of phosphorylated PAK1, PAK1, GFP (for expression of DN-Cdc42 and DN-Rac1) and RhoGDI (loading control). Phosphorylation of PAK1 is dependent on Akt signaling. (**F**) Cells were transfected with DN-PAK1 or pre-treated with a PAK or Akt inhibitor, before treatment with HRG for 30 min and analysis of phosphorylated Stathmin, Stathmin and PAK1 by Western blotting. (**G**) Cells were transfected with EGFP-tubulin, active GSK3 and/or DN-PAK1. The percentage of cells with captured or disassembled microtubules was evaluated.

We have then investigated the signaling pathway by which the PI3K/Akt pathway regulates microtubule stability. GSK3 is a major substrate of Akt. GSK3 is a constitutively active kinase in resting cells and phosphorylation of GSK3β/α on Ser9/21 reduces GSK3 activity. Moreover, GSK3 activity has been associated with microtubule stability during neuron axonal growth [15]. We have thus investigated the possible contribution of GSK3 to PI3K-dependent microtubule stability. We found that inhibition of GSK3, via addition of LiCl or via a dominant negative construct (data not shown), prevented microtubule disassembly induced by inhibition of PI3K/Akt (Fig. 7A,D) and restored oriented migration (Fig. 7B). Our data imply that ErbB2 induces stabilization of microtubules via PI3K/Akt-dependent repression of GSK3 activity.

Microtubule stability is dependent on the balance between stabilizing and destabilizing microtubule-associated proteins (MAPs). We have investigated the implication of Stathmin, a major destabilizing MAP, in ErbB2-dependent microtubule stability. Activation of ErbB2 induced phosphorylation of Stathmin, as seen by the use of an antibody directed against phosphorylated Ser16, one of the main phosphorylation sites of Stathmin (Fig. 7C). Inhibition of PI3K activity strongly decreased ErbB2-induced phosphorylation of Stathmin in both SKBr3 (Fig. 7C) and T47D cells (data not shown). Phosphorylation of Stathmin was previously demonstrated to result in functional inactivation, by decreasing the ability of Stathmin to associate with microtubules [16]. Thus, inhibition of PI3K/Akt could prevent Stathmin inactivation, resulting in Stathmin-mediated microtubule destabilization. We evaluated this hypothesis by depleting Stathmin with siRNAs, before inhibiting the PI3K/Akt pathway. We observed that depletion of Stathmin prevented microtubule destabilization induced by inhibition of PI3K or Akt (Fig. 7A,D) and restored chemotaxis (Fig. 7B), showing that Stathmin is a critical component of the ErbB2/PI3K/Akt pathway that controls microtubule stability. Since inhibition of GSK3 also restored microtubules in PI3K/Akt inhibited cells, we investigated GSK3 acted via the regulation of Stathmin phosphorylation. Concomitant inhibition of PI3K and GSK3 activity did not restore phosphorylation of Stathmin (Fig. 7C) showing that GSK3 does not act via the regulation of Stathmin and suggesting that GSK3 and Stathmin belong to parallel pathways.

Since GSK3 did not regulate the phosphorylation of Stathmin, we investigated other potential regulators of Stathmin. The PAK kinases were good candidates, as they were previously suggested to directly control the phosphorylation of Stathmin downstream of the EGFR [17]. Activation of ErbB2 led to phosphorylation of PAK1/2 (Fig. 7E). Interestingly, PAK phosphorylation was partially reduced by inhibition of Rac1 or Cdc42, but was strongly diminished by inhibition of Akt (Fig. 7E). In addition, we found that inhibiting PAK1 activity, using DN-PAK1 construct or a chemical inhibitor, led to decreased HRG-induced Stathmin phosphorylation (Fig. 7F), suggesting that PI3K/Akt function via PAK-mediated phosphorylation of Stathmin. Thus, the microtubule destabilizing effect of PI3K inhibitors appears to be mediated by the activation of GSK3 on the one hand and the inhibition of PAK1 on the other hand. However, expression of an active form of GSK3 did not result in microtubule disassembly, but induced defective microtubule capture (Fig. 7G). Similarly expression of DN-PAK1 resulted in non-captured microtubules. Interestingly, concomitant expression of active GSK3 and DN-PAK1 significantly increased microtubule disassembly (Fig. 7G), mimicking inhibition of PI3K/Akt.

Thus, our data suggest that ErbB2-induced activation of PI3K/Akt leads to activation of PAK1 and phosphorylation of Stathmin on the one hand and inhibition of GSK3 activity on the other hand, to support microtubule stability and chemotaxis.

## Discussion

**Figure 8:**
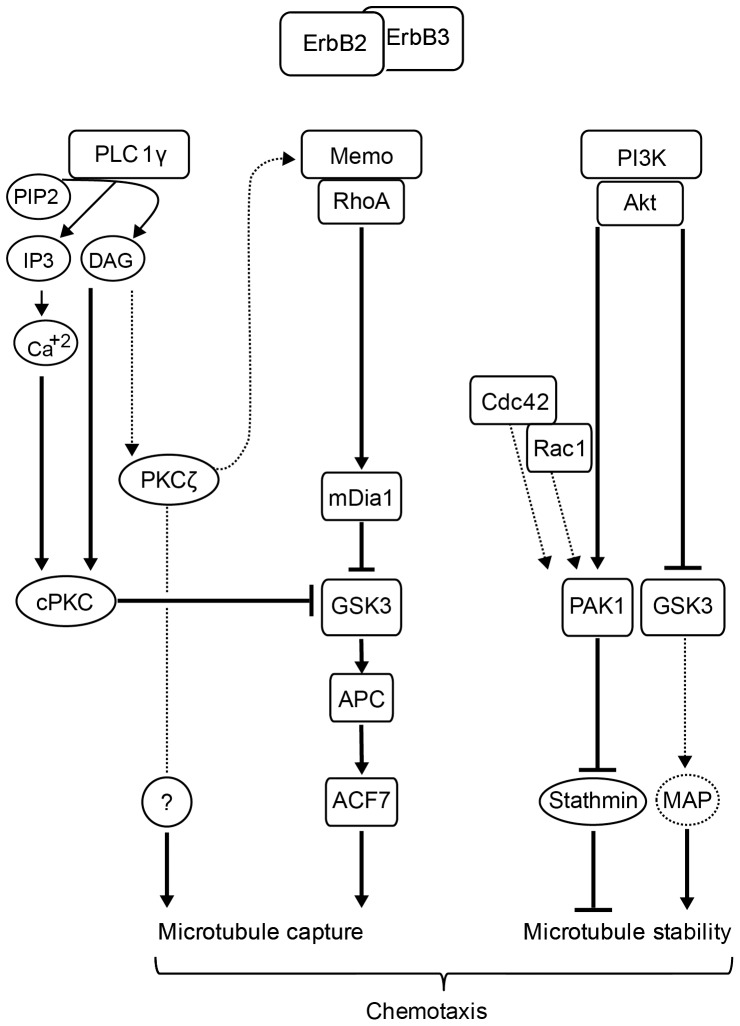
Model of the ErbB2 signaling network that controls microtubule capture, microtubules stability and chemotaxis. Our data show that ErbB2 controls microtubules via distinct, but complementary, signaling pathways. The Memo/RhoA and the PLCγ/cPKC pathways converge to control ACF7 localization at the leading edge, allowing microtubule capture. While PKCζ is also required downstream of PLCγ, its mode of action is still unknown. The PI3K/Akt pathway contributes to microtubule stability, at later stages, and involves the combined effect of the PAK/Stathmin pathway and repression of GSK3 activity. Microtubule capture and stabilization allow the chemotactic response to HRG.

Receptor tyrosine kinases stimulate cell migration via numerous intracellular pathways, including kinase- and GTPase-dependent signaling cascades. It is important to understand how these multiple pathways coordinate in time and space the discrete subcellular processes that underlie cell motility and in particular microtubule dynamics. Microtubules can regulate cell motility in different ways: they contribute to cell asymmetry and the delivery of cargos to the cell front, they control the assembly of the actin cytoskeleton and modulate adhesion site turnover [18]. We have determined how ErbB2-induced signaling pathways are coordinated to control microtubule stabilization at the leading edge. We found that the Memo and the PLCγ-dependent pathways act together to control microtubule capture at the cell front during the early steps of motility, whereas the PI3K/Akt pathway stabilizes microtubules at later times, via the control of Stathmin phosphorylation and GSK3 activity (Fig. 8). Moreover, we observed that microtubule capture and stabilization within cell protrusions determine the ability to migrate along the chemotactic gradient.

PLCγ was shown to control cell chemotactic migration downstream of ErbB2 [7], [13]. We found that the PLCγ and Memo pathways converged toward a single pathway that controlled ACF7 association to the cell leading edge. Our data showed that PLCγ-dependent microtubule capture required the activity of conventional PKCs. PKCs have been involved in microtubule stabilization downstream of mDia2 during wound-healing dependent fibroblast migration [19], even though the study identified nPKCs and excluded conventional cPKCs, as important actors for controlling microtubule stability. Moreover, mDia2/nPKC did not contribute to MTOC reorientation – which was dependent on a distinct Cdc42 signaling complex. In contrast, the Memo/PLCγ/cPKC pathway controlled both microtubule capture at the cell leading edge and cell orientation, in response to HRG.

Interestingly we observed that interfering with individual cPKCs yielded cells with microtubules arrested within the lamella, at a short distance of the leading edge. These cells were capable of chemotactic migration in response to the HRG gradient, suggesting that these microtubules were anchored at a yet undefined structure. A previous study suggested that microtubules were connected to actin filaments, via ACF7, in the vicinity of adhesion sites [20]. More recently it was shown that, in astrocytes, microtubules came in closest contact to the basal plasma membrane at ∼5 µm behind the leading edge, in a region distinct from the actin-rich adhesive zone, thanks to dynein-generated forces [21]. Further investigation should determine if, upon cPKC disturbance, microtubules anchor at the basal plasma membrane, at or behind adhesion-enriched sites, and by what mechanism.

In our work, PKCζ was also found to contribute to microtubule capture downstream of PLCγ, which could appear surprising as aPKC activation is not dependent on Ca^++^ or DAG. However, a recent study showed that DAG conversion into phosphatidic acid, by diacylglycerol kinase α, can provide a signal for recruitment and activation of aPKCs [22]. Interestingly, PKCζ appeared to contribute to the recruitment of Memo and all downstream effectors; moreover, our data also showed that, in contrast to cPKCs, aPKCs did not act solely via the recruitment of ACF7. It will therefore be important to investigate the complex mechanism by which aPKCs contribute to microtubule capture.

Previous studies suggested that establishment of polarity and centrosome repositioning were determined by the Par6/aPKC complex, downstream of Cdc42, via repression of GSK3 activity [23], [24], [25]. Interestingly, in our cellular model, invalidation of aPKCs affected cell orientation along the gradient, without preventing antero-posterior cell polarity, underscoring the fact that the control of cell polarity and the control of cell orientation during motility depend on distinct mechanisms. Thus, aPKCs might stand at the crossroads of pathways directing cell orientation, downstream of PLCγ and cell polarity, downstream of Cdc42.

Cdc42 itself plays a dual role in chemotaxis. Indeed, expression of DN-Cdc42 disturbed cell orientation without disturbing front-rear polarity. However, we observed that when it is expressed at very high levels, DN-Cdc42 induced cell rounding (KB and AB, unpublished data). This observation suggests that low Cdc42 activity might be sufficient to allow cell polarization, and that higher levels of Cdc42 activity are required for cell orientation. This latter function could involve the control of MTOC re-orientation via the recruitment of the minus-end directed dynein motor at cortical sites and generation of pulling forces on microtubules [21], [26], [27].

Memo and PLCγ control the recruitment to the leading edge of cofilin [13], a protein involved in early chemotaxis events, such as localized actin assembly and protrusion [28], [29]. Interestingly, a recent study suggested that in neutrophils, PLCβ/PKC controlled GSK3 phosphorylation and as a consequence cofilin phosphorylation and chemotaxis [30]. We do not know whether this pathway is functional in epithelial cells; but it is tempting to speculate that the PLC/PKC/GSK3 axis might coordinate both actin cytoskeleton reorganization and microtubule capture during chemotaxis.

Our results show that the PI3K/Akt is required to maintain a stable microtubule network during migration. In the absence of PI3K or Akt activities, captured microtubules progressively disassembled and cells lost the ability to follow the chemotactic gradient. Our data show that PI3K functions via the control of Stathmin activity. Several kinases are able to directly phosphorylate Stathmin [31]. Among the few that phosphorylate Stathmin on Ser16, PAK1 was a good candidate, because of its described links with ErbB2 [32], [33]. We confirmed that ErbB2-induced phosphorylation of Stathmin was dependent on PAK activity, which in turn was dependent on PI3K activity. Other reports have involved PI3K signaling in the activation of PAK: Akt phosphorylation of Ser21 and PDK phosphorylation of threonine 423 were found to contribute to PAK activation [34], [35]. But the physiological relevance of these observations has been questioned. We show here that PI3K-dependent activation of PAK has a critical function for the control of Stathmin phosphorylation and microtubule stabilization. It will be interesting to determine how Rac1, Cdc42 and PI3K signals are integrated to regulate ErbB2-induced activation of PAK. We observed a delay between the (early) inhibition of Stathmin phosphorylation by PI3K/Akt and the (late) disassembly of microtubules, suggesting that microtubules might be transiently protected from disassembly by capping proteins or MAPs.

GSK3, a well-known target of Akt, also contributed to microtubule stability, downstream of PI3K. GSK3 activity, however, did not affect Stathmin phosphorylation, showing that PI3K initiated at least two distinct pathways that contributed to microtubule stability. GSK3 might act via the regulation of stabilizing MAPs, as demonstrated in neurons [36], [37]. We do not know what MAPs might be involved downstream of GSK3 in our experimental model, but MAP4 and Stathmin were suggested to have interphase-specific, counteractive and phosphorylation-inactivated activities in many mammalian cell types [38].

Collectively our results underline that distinct and complementary ErbB2-dependent signaling pathways coordinately control microtubule capture and microtubule stability to allow chemotactic migration. Kinases such as GSK3 or PAK1 appear pivotal for these functions. The fact that the same switch kinases are also central for processes such as cell-to-matrix adhesion and actin assembly/disassembly illustrates how signaling pathways controlling different aspects of cell motility are intermingled. Such signaling nodes, which integrate various signaling to coordinate an array of discrete processes, constitute attractive therapeutic targets in the context of migration-related pathologies.

## Materials and Methods

### Cell culture and transfection

SKBr3 and T47D breast carcinoma cells were obtained from ATCC-LGC Standards (Molsheim, France). Cells grown in DMEM and 10% FCS were transfected by nucleofection (Lonza, Basel, Switzerland) with 21-mer siRNAs (synthesized by Life technologies, Saint Aubin, France) targeting Memo (AF132961-1438), ACF7 (NM_012090-3015 and 16814), PLCγ1 (NM_002660-217 and 4968), Stathmin (BC082228.1-378 and 936) and LacZ (M55068-4277) used as negative control; target identifier-first nucleotide of the targeted sequence is indicated between parentheses. Efficiency of siRNAs was verified by Western blotting (Fig. 7 and supplementary material Fig.S1). Efficiency of ACF7 siRNA was verified by flow cytometry (supplementary material Fig.S1). Specificity of siRNAs was verified by using two different siRNAs yielding similar phenotypes. When indicated, cells were also transfected with the following cDNA constructs: EGFP-α-tubulin (Clontech, Saint-Germain-en-Laye, France), Flag-mDia1ΔN3 (S. Narumiya, Kyoto University, Japan), APC (B. Vogelstein, Johns Hopkins Kimmel Cancer Center, Baltimore; Addgene plasmid 16507), GSK3β S9A, GSK3β K85A (J. Woodgett, Mount Sinai Hospital, Toronto; Addgene plasmids 14754/14755) and EGFP-ACF7-NC (E. Fuchs, Rockefeller University, New York). EGFP-ACF7-NC-CCKVL was produced as described previously [11]. Dominant negative constructs for PKCα, PKCβ1, PKCβ2, PKCγ, PKCδ, PKCε, PKCη and PKCζ (Addgene 21235/16381/16385/21239/16389/21243/21246/21249) were from I. B. Weinstein (Columbia University, New York) and Myr-PKCα-Flag and Myr-PKCζ-Flag (Addgene 10807 and 10802) were from A. Toker (Harvard Medical School, Boston).

### Pharmacological agents

HRGβ1 was from R&D Systems (Lille, France). LiCl was from Sigma-Aldrich (Lyon, France) and was used at 20 mM. LY294002 (50 nM); Akt Inhibitor VIII (20 nM); MEK inhibitors, PD098059 (30 µM) and UO126 (50 nM); p38MAPK inhibitor SB203580 (30 nM); cPKC and PKCζ inhibitory peptides (PKCα/β pseudosubstrate and PKCζ pseudosubstrate respectively; 50 nM), and PAK inhibitor IPA-3 (30 µM) were from Merck Chemicals (Nottingham, UK).

### Analysis of microtubules status and immunofluorescence

For microtubules status analysis, cells transfected with EGFP-tubulin were grown on collagen-coated glass coverslips for 48 h and observed upon addition of 5 nM HRGβ1 using the 63× objective (plan apochromat NA 1.4) of a fluorescence microscope (Zeiss Axiovert 200) driven by Metamorph 6.3 software. Images were acquired using a digital camera (Coolsnap HQ; Roper Scientific). At least 200 cells were counted for each condition in three independent experiments. For immunofluorescence, cells grown on collagen-coated coverslips were fixed in 4% paraformaldehyde and permeabilized in 0.2% Triton-X-100 before addition of antibodies directed toward mDia1/DRF1 (Santa Cruz Biotechnology), APC C terminus (Santa Cruz Biotechnology, Heidelberg, Germany) and phospho-GSK3β (Cell Signaling Technology). Alexa secondary antibodies were from Life technologies. When required, signal was amplified using a biotinylated secondary antibody and streptavidin-FITC (BD Biosciences, Le Pont de Claix, France). DNA was counterstained with Hoechst dye (Sigma-Aldrich). Images were recorded with a confocal Imager (Z1 Zeiss microscope), 100× plan objective (1.4 NA) coupled to an AxioCam MRm camera, driven by LSM software. The percentage of cells displaying cortical labeling was quantified. More than 300 cells were counted for each condition in three independent experiments. Results were analyzed with GraphPad Prism. Means and SEM are shown.

### Chemotaxis assay

Chemotaxis was measured by direct observation and recording of cell behavior in a stable HRGβ1 gradient using Dunn chemotaxis chambers (Hawksley Technology, Lancing, UK) as described [39], [40]. Cells were grown on collagen-coated glass coverslips for 48 hours before setting up the chemotaxis chambers. Gradients of HRGβ1 were formed by placing HRGβ1-containing (10 and 40 nM for SKBr3 and T47D cells respectively) DMEM/10% FCS in the outside well and HRGβ1-free DMEM/10% FCS in the inside well. For no gradient controls, DMEM/10% FCS with 5 and 10 nM HRGβ1 for SKBr3 and T47D cells was placed in both the inside and outside wells. Images were acquired using the 10× plan apochromat (NA 1.4) objective of a Zeiss Axiovert 200 microscope with a time interval of 8 minutes for 8 and 4 hours for SKBr3 and T47D cells, respectively, using a digital camera (Coolsnap HQ; Roper Scientific) driven by Metamorph 6.3 software. 90 to 150 cells were tracked for each condition in three independent experiments using Metamorph 6.3 software. Tracks were analyzed using the Chemotaxis and Migration Tool (Image J) to obtain cell speed and persistence (ratio of total distance travel to Euclidian distance). Statistical analysis was performed with GraphPad Prism. Means and SEM are shown. Cell orientation was shown as Rose plots. The Rayleigh test for unimodal clustering of directions was used to test if the cell population displayed directionality in the chemotactic gradient [39]. Distribution of directions was considered uniform (random migration) for p>0.05.

### Western Blotting

Cells were collected in lysis buffer as described [10]. Western blotting was performed using antibodies directed against Memo (monoclonal antibody to amino acids 25–43), PLCγ1, GSK3β, phospho-GSK3β (Ser9), phospho-PAK (Cell Signaling Technology, Danvers, MA, USA), mDia1, Cdc42, RhoGDIα, PAK, HA (Santa Cruz Biotechnology), α-tubulin, Stathmin (Sigma-Aldrich), Rac1 (BD Transduction Laboratories) and phospho-Stathmin (Ser16) (a kind gift from A. Sobel, Institut du Fer à Moulin, Paris).

### Tubulin/microtubule fractions

Cells were grown for 48 h before treatment with the indicated drug for 1 h, and then stimulated with HRG β1. Cells were washed twice with warm phosphate buffer saline, before addition of warm OPT buffer (80 mM K-PIPES pH 6.8, 1 mM EGTA, 1 mM MgCl_2_, 0.5% Triton-X-100, 10% glycerol containing complete protease inhibitor cocktail from Roche and phosphatase inhibitors cocktails II and III from Sigma-Aldrich). The dish was stirred very gently by hand and the soluble fraction (containing “soluble tubulin”) immediately recovered. Cells were washed once with warm OPT before scrapping in OPT buffer and freezing at −80°C. Samples were centrifuged at 10 000 g, before collecting the supernatant (“microtubule fraction”).

## Supporting Information

Figure S1 **Validation of Memo, ACF7 and PLCγ1 siRNAs.** SKBr3 cells were transfected with siRNAs or an ACF7-CCKVL construct, as indicated. Expression of the target protein was analyzed by Western Blotting (**A and C**). An anti-RhoGDI antibody was used to assess comparable protein loading. Expression of ACF7 was analyzed by FACS (**B**) as described before (11). A combination of both PLCγ1 siRNAs was used in this study.
(TIF)

Figure S2 **PLCγ1 and Memo signaling converge downstream of mDia1 and upstream of GSK3.** Still images of EGFP-tubulin expressing cells migrating in response to HRG, analyzed by time-lapse fluorescence microscopy 30 min after addition of HRG. Cells were transfected with Memo or PLCγ1 siRNA, together with active mDia1, DN-GSK3, ACF7 minigene (ACF7) or membrane-targeted ACF7 minigene (ACF7-CCKVL). Insets: zooms the presence or absence of peripheral microtubules in cell protrusions. Quantification is shown in Fig. 3C.
(TIF)

Figure S3 **Impact of Memo or PLCγ1 depletion on leading edge recruitment of mDia1, phosphorylated GSK3 (P-GSK3) and APC.** SKBr3 cells expressing control, Memo, or PLCγ1 siRNA, were treated with 5 nM HRGβ1 for 20 min and processed for immunofluorescence using antibodies against mDia1, P-GSK3 or APC. Right panels show the percentage of cells with leading edge labeling: 90–150 cells were counted per condition in three independent experiments, mean+/−s.e.m. is shown; * p<0.01. PLCγ depletion affects localization of P-GSK3 and APC but not of mDia1, confirming that PLCγ, in contrast to Memo, acts downstream of mDia1.
(TIF)

Figure S4 **Role of PKCs in microtubule capture.** SKBr3 cells were transfected with EGFP-tubulin and the indicated constructs 48 h before analysis. Expression of the different HA-tagged PKC constructs was verified by Western blotting using an anti-HA antibody (upper panel). Impact of the different constructs on microtubule capture was quantified as in Fig. 4 (lower panels). 90–150 cells were counted per condition in three independent experiments, mean+/−s.e.m. is shown; * p<0.01.
(TIF)

Figure S5 **PKCs contribute to PLCγ-dependent microtubule capture and chemotaxis.** Still images of EGFP-tubulin expressing cells migrating in response to HRG, analyzed by time-lapse fluorescence microscopy 30 min after addition of HRG. (**A**) Cells expressing APC or membrane-targeted ACF7 (ACF-CCKVL) were treated with PKC inhibitory peptides, before addition of HRG. Expression of APC or ACF7-CCKVL only rescues microtubules of cPKC-inhibited and not of aPKC-inhibited cells. (**B**) SKBr3 cells were transfected with control (Ctrl) or PLCγ siRNA and constitutively active PKCα (myr-PKCα) or PKCζ (myr-PKCζ). Active PKCζ stabilizes microtubules at the leading edge, while active PKCα stabilizes microtubules at a short distance of the leading edge. Quantification is shown in Fig. 5.
(TIF)

Figure S6 **Impact of different type of PKCs on leading edge recruitment of mDia1, phosphorylated GSK3 (P-GSK3) and APC.** (**A**) SKBr3 cells expressing DN-PKC constructs or pretreated with cPKC inhibitory peptide were treated with 5 nM HRGβ1 for 20 min and processed for immunofluorescence using antibodies against mDia1, P-GSK3 or APC. Right panels show the percentage of cells with leading edge labeling. (**B**) Cells expressing control vector or DN-PKCζ and/or EGFP-RhoA were labeled with antibodies to Memo, mDia1, P-GSK3 or APC. 90–150 cells were counted per condition in three independent experiments, mean+/−s.e.m. is shown; * p<0.01.
(TIF)

Figure S7 **Role of ErbB2-induced canonical pathways in microtubule stability and chemotaxis.** SKBr3 cells expressing EGFP-α tubulin were pretreated with inhibitors against PI3K, Akt, MEK and p38MAPK for 60 min, before addition of HRGβ1 for 90 min and analysis by time-lapse fluorescence microscopy. (**A**) Cells were assayed for chemotaxis in response to HRG. Rose diagrams and Rayleigh tests are shown. (**B**) Still images are shown. (**C**) The percentage of cells with disassembled microtubules was evaluated. 90–150 cells were counted per condition in three independent experiments, mean+/−s.e.m. is shown; * p<0.01. (**D**) **Both GSK3 activity and Stathmin contribute to microtubule stability**. LY294004 was added to Stathmin siRNA-expressing cells or to cells treated with LiCl, before addition of HRGβ1. Still images are shown.
(TIF)

Video S1 **Inhibition of PI3K leads to microtubule disassembly.** SKBr3 cells expressing EGFP-tubulin α were grown on collagen-coated glass coverslips. Cells were then pre-treated with solvent (**left**) or the PI3K inhibitor LY294004 (**right**) for 1 h, before addition of 5 nM HRGβ1 and observation using the 63× objective (plan apochromat NA 1.4) of a fluorescence microscope (Zeiss Axiovert 200). Images were acquired every 4 min for 90 min using a Coolsnap HQ digital camera. Microtubules are stable over the time of the experiment in the control cells, while microtubules are disassembled starting ∼30 min after HRG addition when PI3K is inhibited. Corresponds to Fig. 6A.
(MOV)
