# Supplementary figures and images for: ErbB2-Dependent Chemotaxis Requires Microtubule Capture and Stabilization Coordinated by Distinct Signaling Pathways
Source: PLoS One. 2013 Jan 29;8(1):e55211. doi: 10.1371/journal.pone.0055211 (PMC3558493; doi:10.1371/journal.pone.0055211)

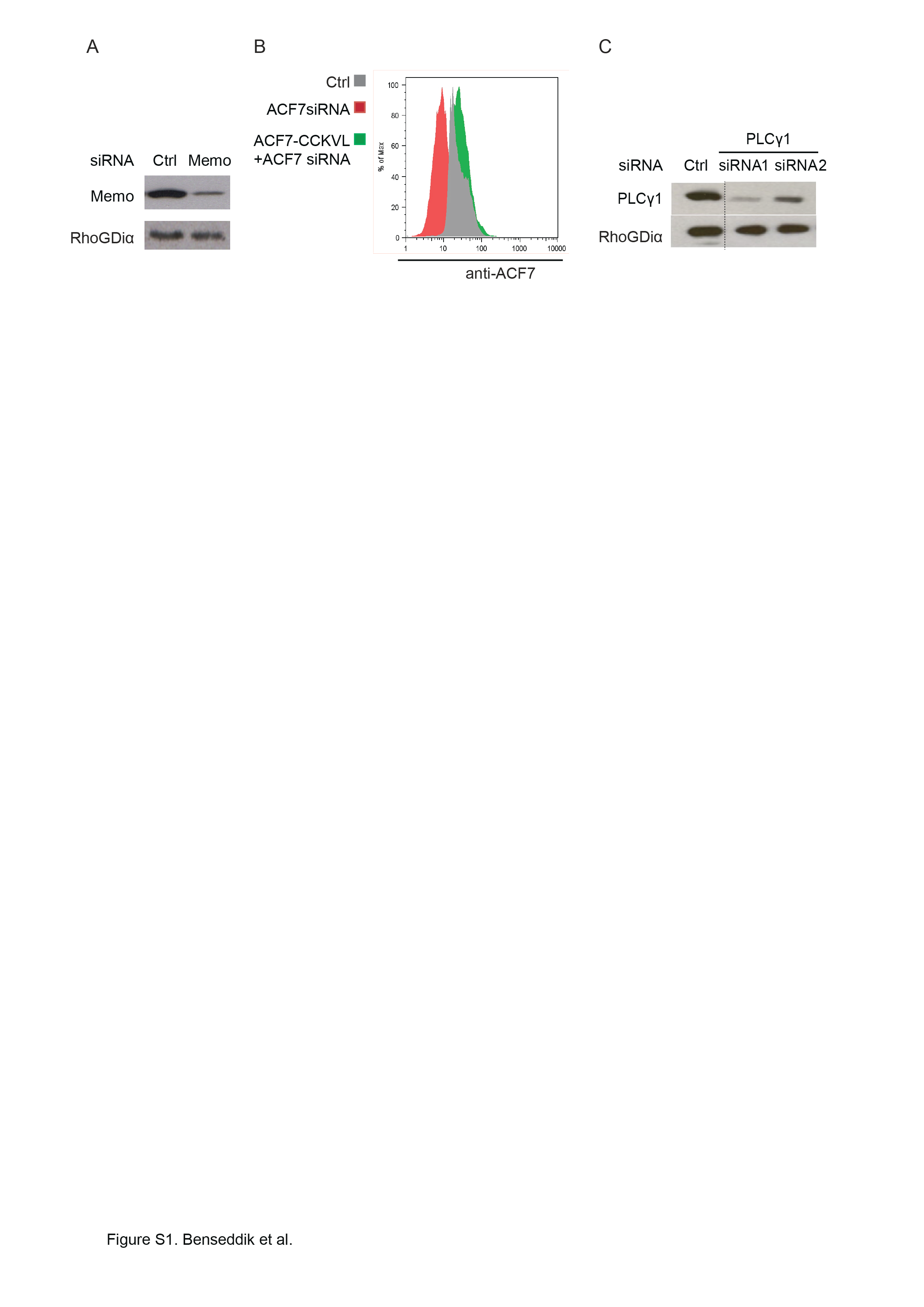

Supplement: Figure S1 — Validation of Memo, ACF7 and PLCγ1 siRNAs. SKBr3 cells were transfected with siRNAs or an ACF7-CCKVL construct, as indicated. Expression of the target protein was analyzed by Western Blotting (A and C). An anti-RhoGDI antibody was used to assess comparable protein loading. Expression of ACF7 was analyzed by FACS (B) as described before (11). A combination of both PLCγ1 siRNAs was used in this study. (TIF) [file pone.0055211.s001.tif]

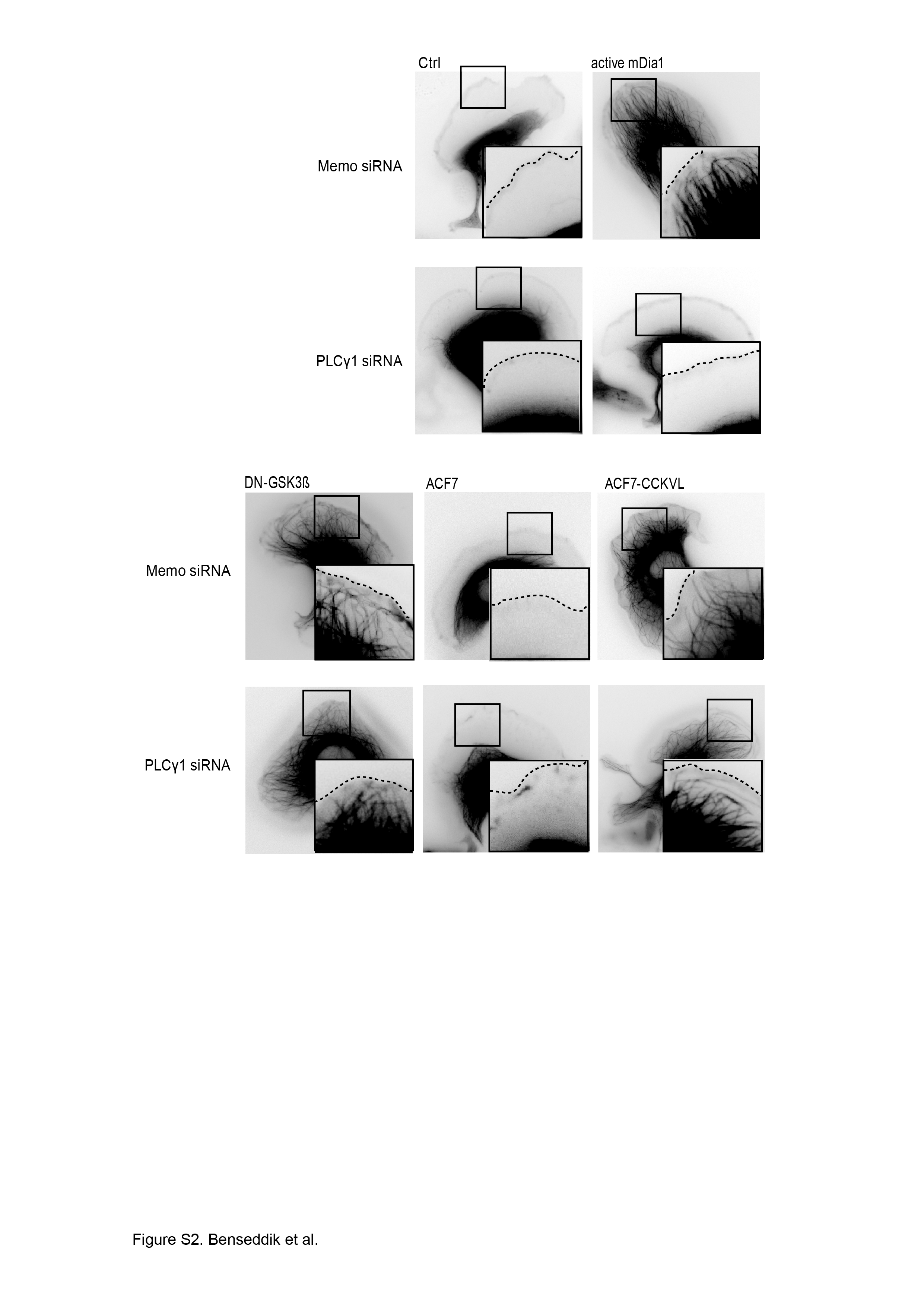

Supplement: Figure S2 — PLCγ1 and Memo signaling converge downstream of mDia1 and upstream of GSK3. Still images of EGFP-tubulin expressing cells migrating in response to HRG, analyzed by time-lapse fluorescence microscopy 30 min after addition of HRG. Cells were transfected with Memo or PLCγ1 siRNA, together with active mDia1, DN-GSK3, ACF7 minigene (ACF7) or membrane-targeted ACF7 minigene (ACF7-CCKVL). Insets: zooms the presence or absence of peripheral microtubules in cell protrusions. Quantification is shown in Fig. 3C. (TIF) [file pone.0055211.s002.tif]

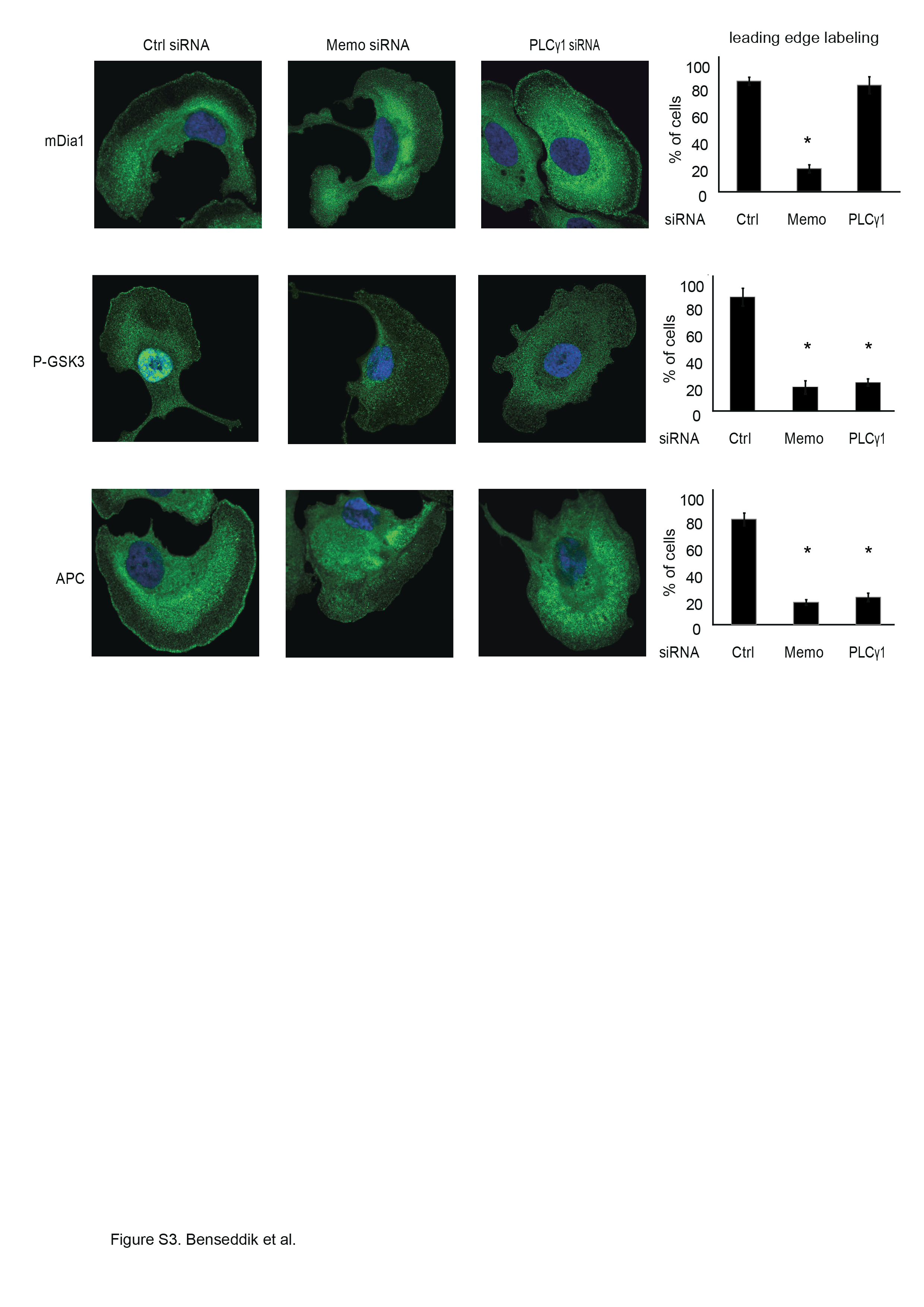

Supplement: Figure S3 — Impact of Memo or PLCγ1 depletion on leading edge recruitment of mDia1, phosphorylated GSK3 (P-GSK3) and APC. SKBr3 cells expressing control, Memo, or PLCγ1 siRNA, were treated with 5 nM HRGβ1 for 20 min and processed for immunofluorescence using antibodies against mDia1, P-GSK3 or APC. Right panels show the percentage of cells with leading edge labeling: 90–150 cells were counted per condition in three independent experiments, mean+/−s.e.m. is shown; * p<0.01. PLCγ depletion affects localization of P-GSK3 and APC but not of mDia1, confirming that PLCγ, in contrast to Memo, acts downstream of mDia1. (TIF) [file pone.0055211.s003.tif]

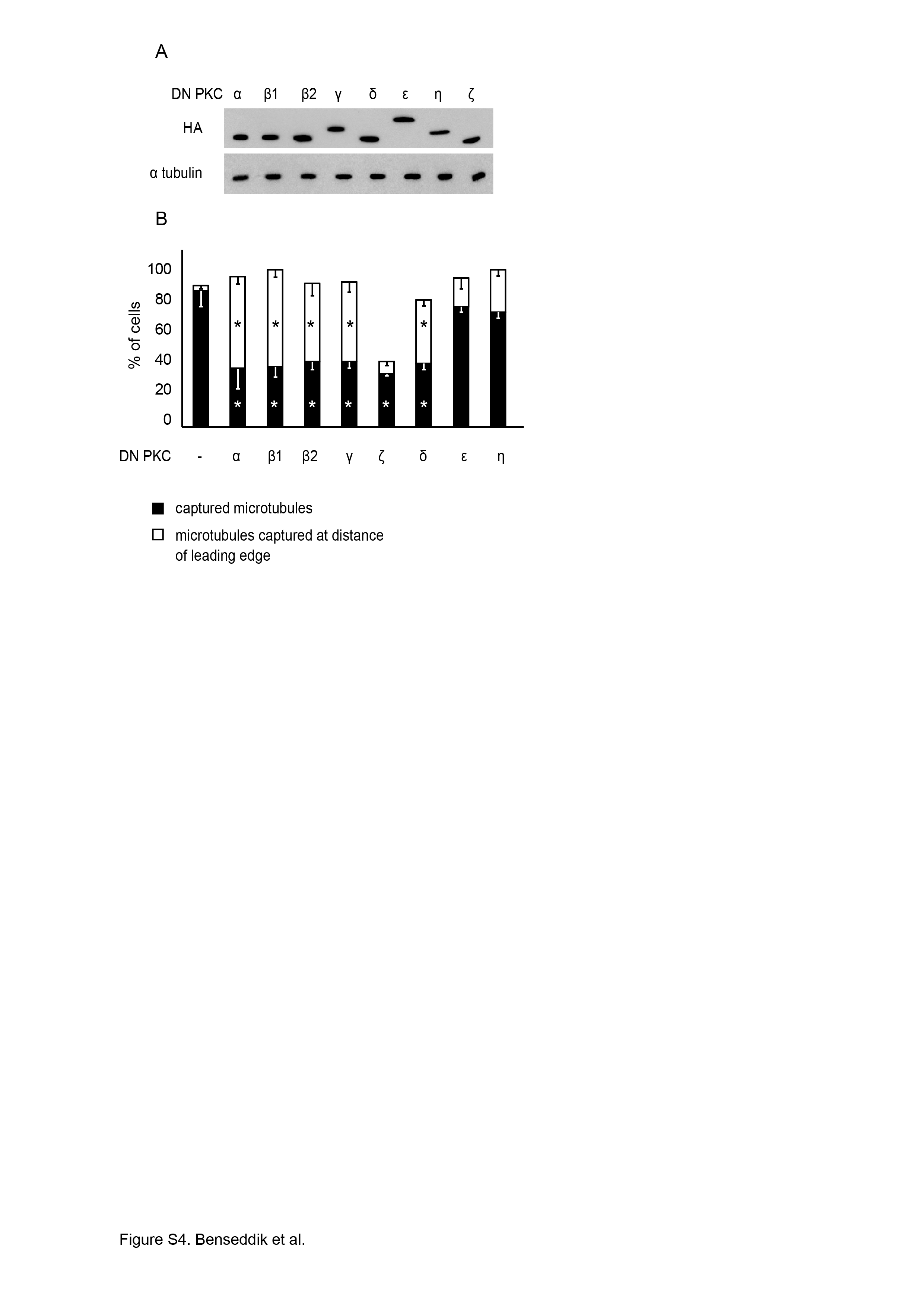

Supplement: Figure S4 — Role of PKCs in microtubule capture. SKBr3 cells were transfected with EGFP-tubulin and the indicated constructs 48 h before analysis. Expression of the different HA-tagged PKC constructs was verified by Western blotting using an anti-HA antibody (upper panel). Impact of the different constructs on microtubule capture was quantified as in Fig. 4 (lower panels). 90–150 cells were counted per condition in three independent experiments, mean+/−s.e.m. is shown; * p<0.01. (TIF) [file pone.0055211.s004.tif]

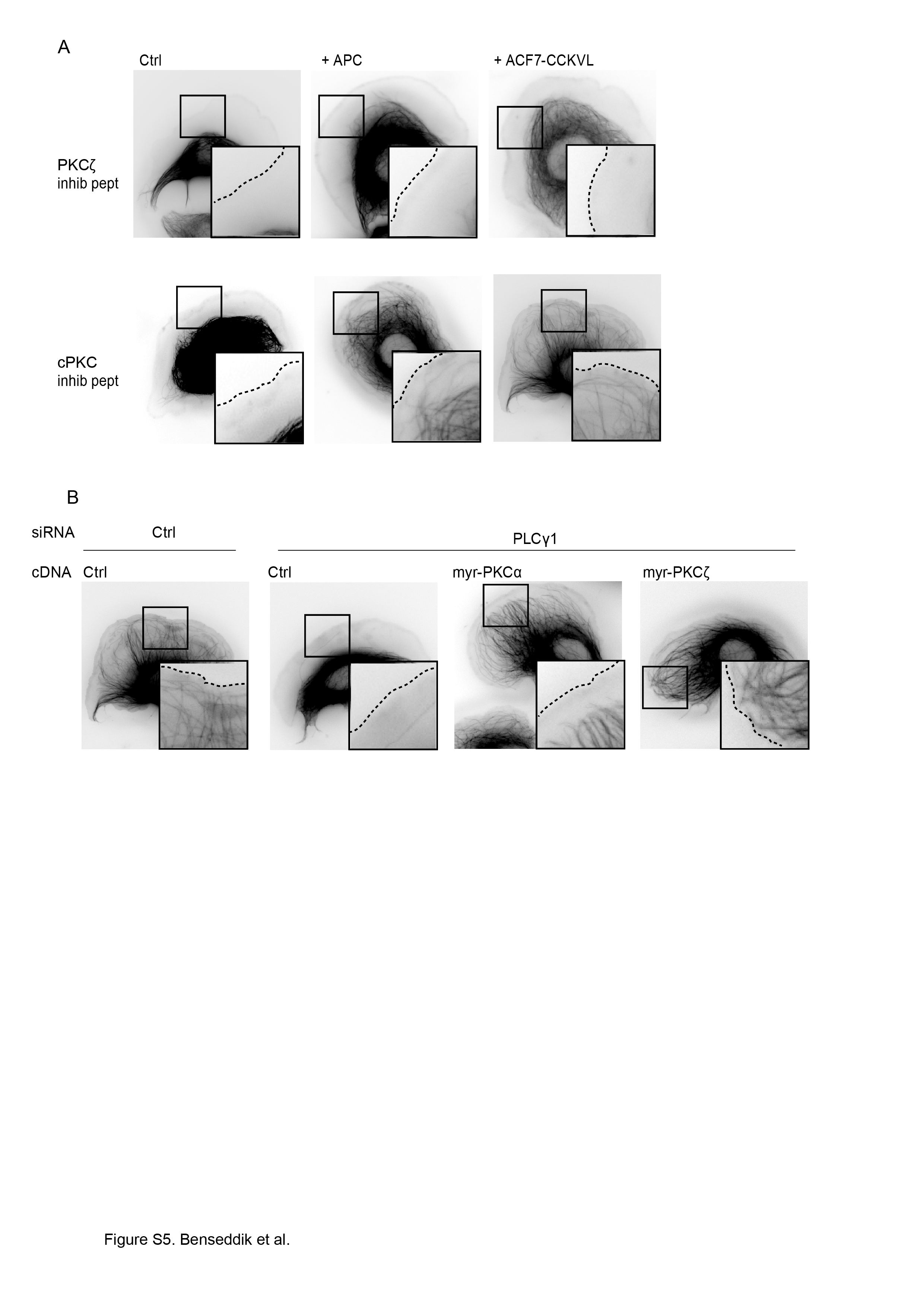

Supplement: Figure S5 — PKCs contribute to PLCγ-dependent microtubule capture and chemotaxis. Still images of EGFP-tubulin expressing cells migrating in response to HRG, analyzed by time-lapse fluorescence microscopy 30 min after addition of HRG. (A) Cells expressing APC or membrane-targeted ACF7 (ACF-CCKVL) were treated with PKC inhibitory peptides, before addition of HRG. Expression of APC or ACF7-CCKVL only rescues microtubules of cPKC-inhibited and not of aPKC-inhibited cells. (B) SKBr3 cells were transfected with control (Ctrl) or PLCγ siRNA and constitutively active PKCα (myr-PKCα) or PKCζ (myr-PKCζ). Active PKCζ stabilizes microtubules at the leading edge, while active PKCα stabilizes microtubules at a short distance of the leading edge. Quantification is shown in Fig. 5. (TIF) [file pone.0055211.s005.tif]

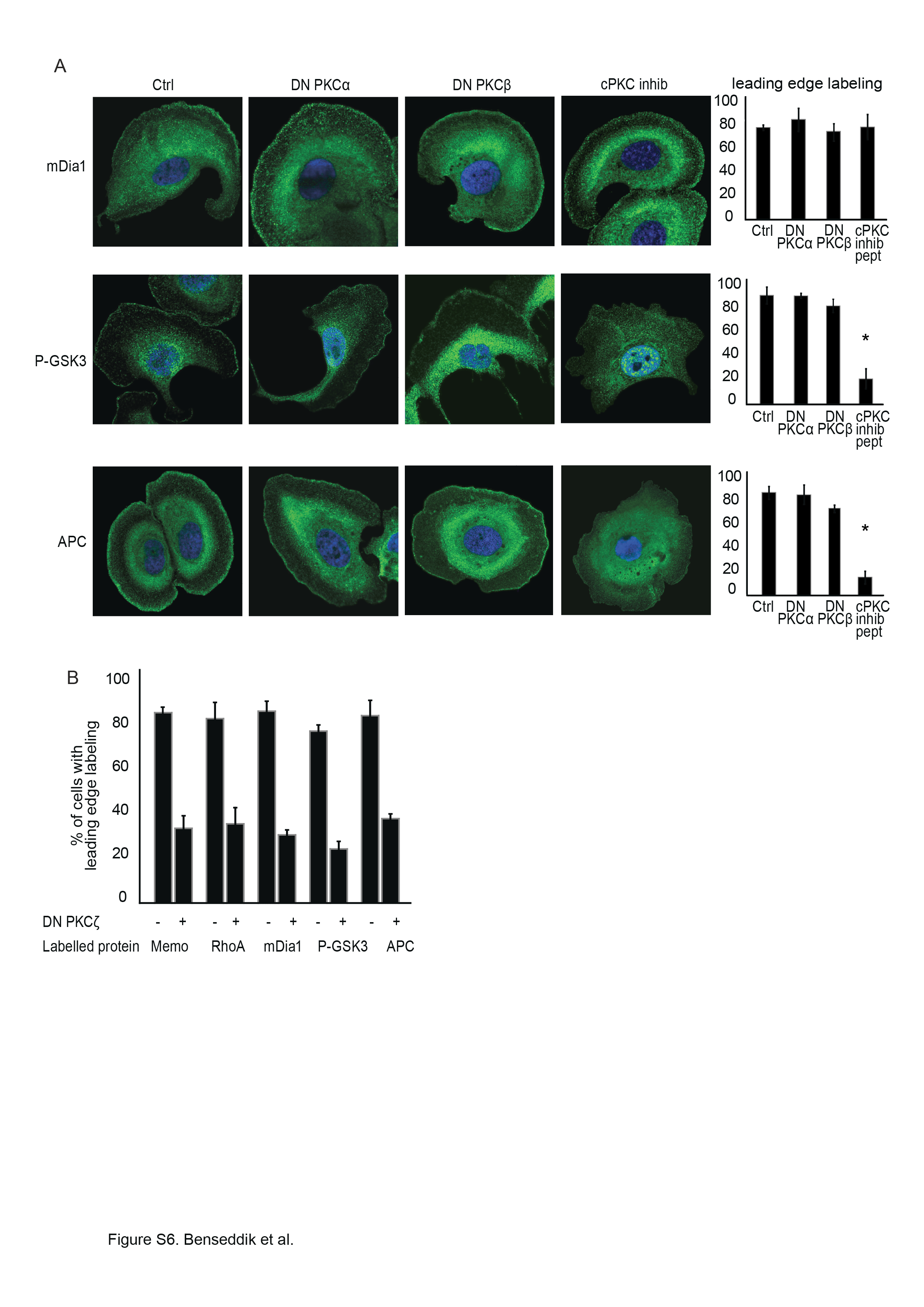

Supplement: Figure S6 — Impact of different type of PKCs on leading edge recruitment of mDia1, phosphorylated GSK3 (P-GSK3) and APC. (A) SKBr3 cells expressing DN-PKC constructs or pretreated with cPKC inhibitory peptide were treated with 5 nM HRGβ1 for 20 min and processed for immunofluorescence using antibodies against mDia1, P-GSK3 or APC. Right panels show the percentage of cells with leading edge labeling. (B) Cells expressing control vector or DN-PKCζ and/or EGFP-RhoA were labeled with antibodies to Memo, mDia1, P-GSK3 or APC. 90–150 cells were counted per condition in three independent experiments, mean+/−s.e.m. is shown; * p<0.01. (TIF) [file pone.0055211.s006.tif]

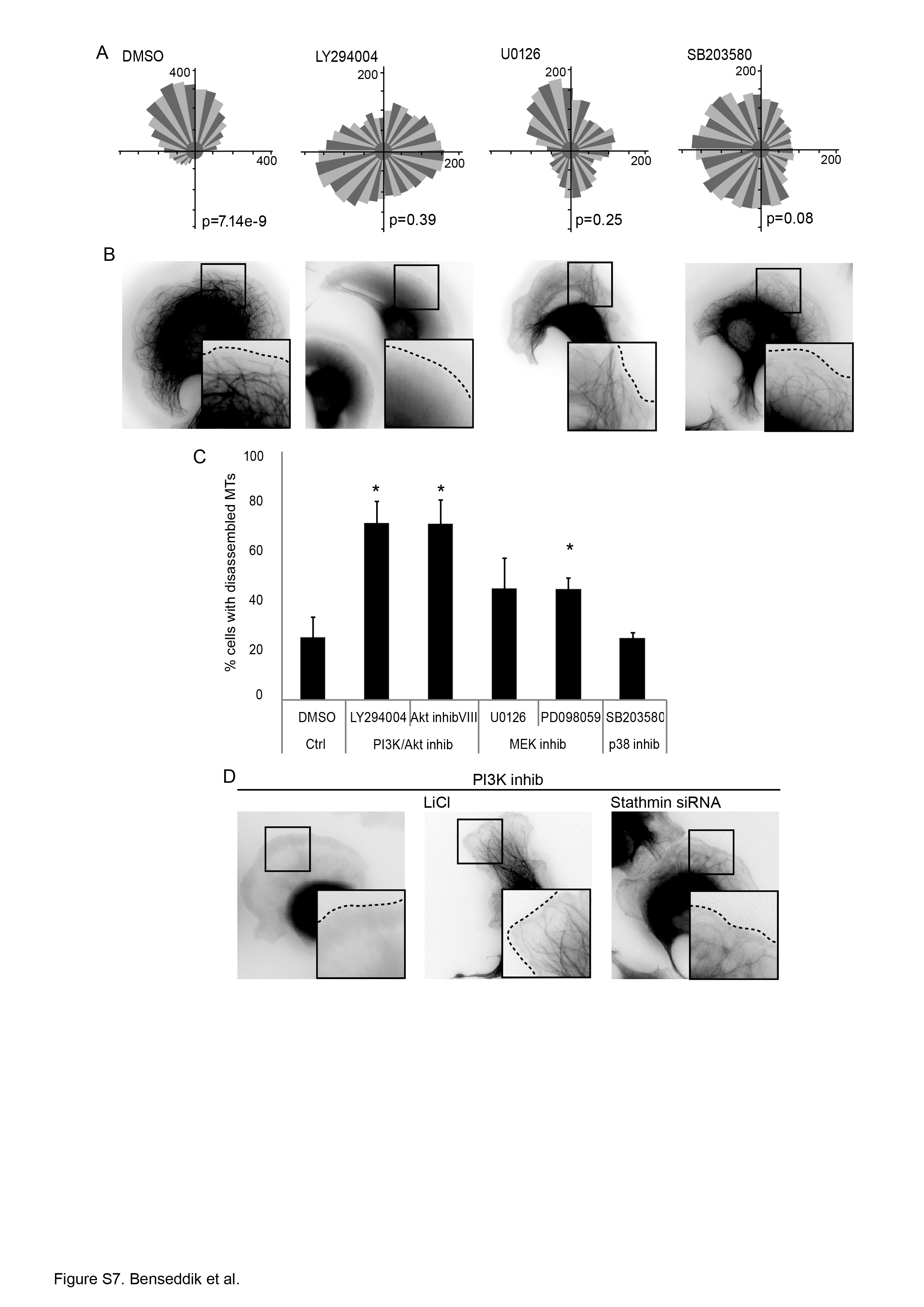

Supplement: Figure S7 — Role of ErbB2-induced canonical pathways in microtubule stability and chemotaxis. SKBr3 cells expressing EGFP-α tubulin were pretreated with inhibitors against PI3K, Akt, MEK and p38MAPK for 60 min, before addition of HRGβ1 for 90 min and analysis by time-lapse fluorescence microscopy. (A) Cells were assayed for chemotaxis in response to HRG. Rose diagrams and Rayleigh tests are shown. (B) Still images are shown. (C) The percentage of cells with disassembled microtubules was evaluated. 90–150 cells were counted per condition in three independent experiments, mean+/−s.e.m. is shown; * p<0.01. (D) Both GSK3 activity and Stathmin contribute to microtubule stability. LY294004 was added to Stathmin siRNA-expressing cells or to cells treated with LiCl, before addition of HRGβ1. Still images are shown. (TIF) [file pone.0055211.s007.tif]
